# Looking at Aesthetic Emotions in Advertising Research Through a Psychophysiological Perspective

**DOI:** 10.3389/fpsyg.2020.553100

**Published:** 2020-09-30

**Authors:** Mathieu Lajante, Olivier Droulers, Christian Derbaix, Ingrid Poncin

**Affiliations:** ^1^Department of Marketing Management, Ted Rogers School of Management, Ryerson University, Toronto, ON, Canada; ^2^CNRS, CREM UMR 6211, University of Rennes 1, Rennes, France; ^3^FUCaM, Catholic University of Louvain, Mons, Belgium; ^4^Louvain School of Management, LouRIM, Université Catholique de Louvain, Mons, Belgium

**Keywords:** advertising, consumer neuroscience, emotion, psychophysiology, cognitive appraisal, skin conductance, facial EMG, aesthetics

## Abstract

Do usual commercials elicit the full spectrum of emotions? For this perspective paper, we posit that they do not. Concepts and measures related to the adaptive functions and well-being areas of emotion research cannot simply be transferred for use in advertising research. When a commercial elicits emotions, the emotions staged in the commercial must not be directly associated with the emotions felt by consumers when exposed to those commercials. This is why “aesthetic” emotions seem more appropriate than “utilitarian” emotions in advertising research, with the former generally felt more significantly than they are acted upon. Aesthetic emotions elicit limited physiological change, and they rely on the intrinsic pleasantness appraisal of commercials. Accordingly, pleasure and displeasure—as observed through expressive and subjective components of aesthetic emotion—often form the first and only step of commercial appraisal, and they are directed toward attitude formation rather than overt behaviors. Our preliminary psychophysiological study shows this by investigating the contributions of psychophysiological and self-reported measures of aesthetic emotions induced by commercials to explain attitudes toward advertisements. The results show that only two components of aesthetic emotion positively influenced attitudes toward the advertisements: expressive (measured by facial electromyography) and subjective (measured by the self-assessment manikin scale). Also, the subjective component of aesthetic emotion partially mediates the effects of the expressive components on attitudes toward the ads. Our exploratory study illustrates the relevance of focusing on aesthetic emotions in advertising research. It also shed new light on the contributions of the physiological, expressive, and subjective feelings components of aesthetic emotions in advertising effectiveness.

## Consumer Emotion in Advertising Research: Why Does It Matter?

Studies in advertising research have investigated the effect of consumer’s emotion on attitude toward the ad (Aad), attitude toward the brand, and purchase intention (e.g., Batra and Ray, 1986; Aaker et al., 1988; Derbaix, 1995). These studies implicitly assumed that commercials can elicit the full spectrum of emotions. We disagree with that assumption. First, concepts and measures related to the adaptive functions and well-being areas of emotion research cannot simply be transferred to advertising research (Scherer and Zentner, 2001, 2008). Second, the emotions elicited by the affect-laden events depicted in commercials should not be confused with the emotions felt by consumers passively exposed to the commercials. Hyundai’s 2016 Super Bowl commercial featuring “talking bears” illustrates our point. In this commercial, the bears attack hikers in a forest who find refuge in their car at the last second thanks to the new Hyundai remote start feature. For hikers, such a situation elicits “high-intensity emergency reactions involving a synchronization of many organismic subsystems,” (Scherer, 2004, p. 241). This refers to utilitarian emotions. However, non-hiking consumers passively exposed to this commercial are not targeted by bear attacks. Actually, they are more likely to feel emotions that are oriented toward experiential phenomena rather than those “in the service of behavioral readiness” (Scherer, 2004, p. 244). This refers to aesthetic emotions.

For this perspective paper, we assume that consumers passively exposed to commercials experience *aesthetic* rather than *utilitarian* emotions.

## Theoretical Background and Specific Expectations

The cognitive appraisal theories (Arnold, 1960; Lazarus, 1982; Moors et al., 2013) state that the nature of emotion is determined by a cognitive appraisal. Emotions are extracted from appraisal of events that cause specific reactions in different people (Scherer and Zentner, 2001); it is not the events themselves that trigger an emotion but the way in which we interpret them (Haimerl, 2008).

Most of these appraisal processes are assumed to be automatic. Leading appraisal theorists postulate that cognitive appraisal is a precondition for emotion but do not equate appraisal with conscious cognition and place the cognitive component at the very onset of the emotional episode (Moors, 2009, p. 638). For example, work in neuroscience has shown that the relationship between cognitive appraisal and emotion is characterized by a cerebral process that simultaneously deals with emotional and cognitive functions (Fugate et al., 2011). Therefore, the distinction between appraisal and emotion is impossible to make consciously by individuals (Barrett et al., 2007, p. 386).

Within the framework of cognitive appraisal theories of emotion, the Component Process Model was developed with the aim of predicting the determinants of emotional episodes and to understand the cognitive mechanisms involved in the development of behavioral readiness (Scherer, 2009). In this model, the triggering of emotion and the determination of its characteristics rely on the subjective, continuous and recursive appraisal of an event perceived as relevant to the individual’s goals. This appraisal process relies on the sequential evaluation of four main criteria—goal relevance, implication, coping, and normative meaning—which will result in physiological, expressive, and subjective responses at the origin of the individual’s behavioral readiness (Scherer, 2009).

Goal relevance is the first step of stimulus appraisal process and plays a central role in determining the intensity of the subsequent emotional episode (Scherer, 1984; Frijda, 1986): “The more important the goal at stake, the stronger the ensuing emotion” (Moors, 2009, p. 640). That’s why in our example, *utilitarian* emotions felt by hikers’ can be stronger than the *aesthetic* emotions experienced by viewers. Utilitarian emotions are those emerging from the interpretation of events having important consequence for the wellbeing: “because of their importance for survival and wellbeing, many utilitarian emotions are high intensity emergency reactions” (Scherer, 2005, p. 706). But this is not the case for most of the emotions experienced when watching commercials.

Contrary to utilitarian emotions, aesthetic emotions “are triggered in situations that usually have no obvious material effect on the individual’s well-being and only rarely lead to specific goal-oriented responses” (Scherer and Zentner, 2008, p. 596). According to Frijda and Sundararajan (2007, p. 232), aesthetic emotions “are more felt than acted upon and thus do not obviously manifest themselves in overt behaviors; [… they] may not show pronounced physiological upset [and] are often about complex events or subtle events aspects.” However, this does not mean that aesthetic emotions are disembodied. Visual and auditory stimuli can induce changes in the consumer, either autonomic (i.e., activation of the autonomic nervous system) or expressive (i.e., activation of the somatic nervous system); therefore, they can define a specific emotional pattern through an appraisal process. For instance, studies have shown that watching commercials arouse both autonomic and expressive components of emotion (e.g., Aaker et al., 1986; Hazlett and Hazlett, 1999).

Aesthetic emotions are more reactive than proactive, and autonomic and expressive changes are primarily based on “the appreciation of the intrinsic qualities” of the stimulus (Scherer, 2004). For Kant (2001), aesthetic experience is disinterested pleasure highlighting the complete absence of utilitarian considerations (referenced by Scherer, 2005, p. 706). Frijda and Sundararajan (2007, p. 236) also suggest that pleasure and displeasure “often form the first step of appraisal, and on occasion they may form the only step” for eliciting aesthetic emotions. In other words, aesthetic emotions rely on an intrinsic pleasantness appraisal that directly affects the expressive component of emotion [e.g., facial electromyography (EMG) responses]. Even though autonomic responses (e.g., skin conductance responses) may be noticeable during aesthetic emotional episodes, they are not oriented toward adaptive action tendencies and display low amplitudes (see Scherer and Zentner (2008) for a discussion of empirical evidence).

In addition to the autonomic and expressive components, subjective feelings are another major component for investigating aesthetic emotions in advertising research. Within the framework of cognitive appraisal theories, subjective feelings represent a central component of emotion that serves as the basis for the conscious representation of emotional processes (“the subjectively experienced feelings of emotion”; Zentner et al., 2008, p. 497). Therefore, we assume that aesthetic emotions induced by commercials “should be studied as (more or less conscious) feelings that integrate cognitive and physiological effects” (Scherer, 2004, p. 239). This is consistent with Aaker et al. (1988) who acknowledged that consumers’ subjective feelings would be more appropriate for investigating advertising effectiveness.

To support our claim that usual commercials elicit aesthetic rather than utilitarian emotions, we investigated the structure of aesthetic emotional episodes and their active components during passive exposure to usual TV commercials. Usual TV commercials refer to ads with pleasant scenes, happy people, witty communication that characterize much of the advertising.

Even though autonomic (e.g., Aaker et al., 1986), expressive (e.g., Hazlett and Hazlett, 1999), and subjective (Aaker et al., 1988) components of aesthetic emotion have been studied in advertising research, it is noteworthy that there is no attempt to investigate the respective contribution of these coexisting components to the formation of subsequent attitudes. Therefore, we proposed an evaluation of the predictive power of those components on attitude toward the ad (Aad). Our specific expectations were the following:

1. Autonomic responses (skin conductance responses) induced by usual TV commercials do not influence Aad.
2. Expressive responses (facial EMG responses) induced by usual TV commercials do influence Aad: Increased activity in the cheek region has a positive effect on Aad, whereas increased activity in the brow region has a negative effect on Aad.
3. Subjective responses (subjective feelings component) induced by usual TV commercials have a positive effect on Aad.

As previously outlined, subjective feelings represent a central component of emotion that integrates all of the underlying emotional processes (Grandjean and Scherer, 2008). Accordingly, we expected that the expressive responses induced by usual TV commercials (but not autonomic responses) would be powerful predictors of subjective responses that in turn would be powerful predictors of Aad.

## A Preliminary Psychophysiological Investigation of Aesthetic Emotions Elicited by Commercials

**FIGURE 1:**
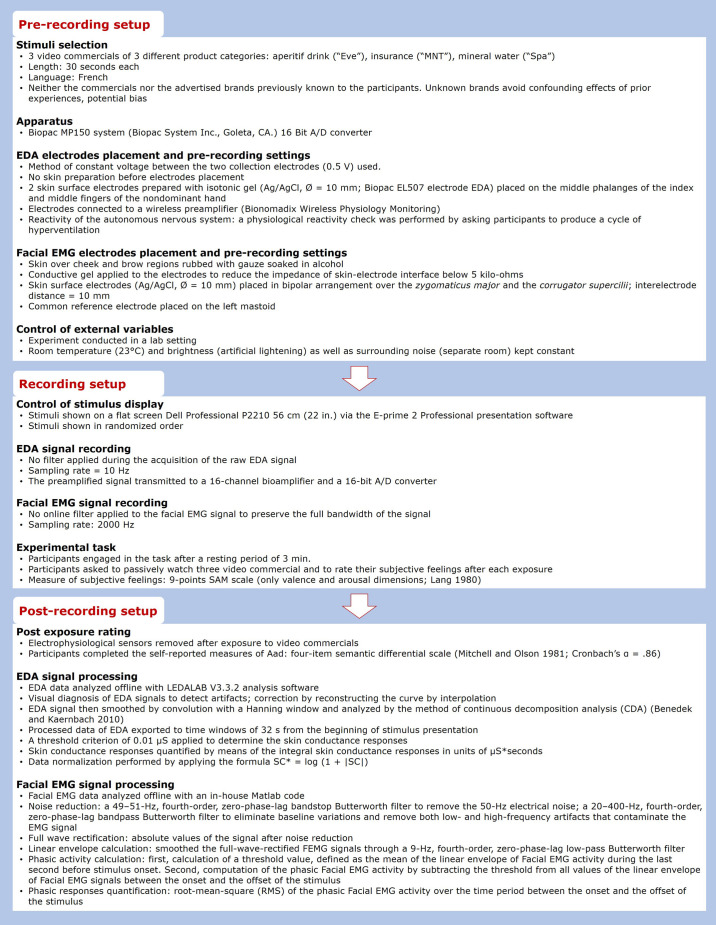
Experimental setup of the psychophysiological study. The figure presents the settings before, during and after the signal recording of both electrodermal activity and facial EMG.

To test our main hypothesis, we conducted a psychophysiological experiment during which 51 voluntary participants (21 female and 30 male) aged between 21 and 25 years (*M* = 23.06, SD = 1.25) were exposed to three video commercials (within-subject design).^1^ Psychophysiological measurements of aesthetic emotion components (autonomic and expressive) were conducted in accordance to the methodological standards and the conditions to design and implement psychophysiological studies (Lajante et al., 2012, 2017; Lajante and Ladhari, 2019; Lajante and Lux, 2020; see Figure 1). Five respondents were eliminated because of a lot of missing values across items or identical responses to all items (self-reported measures) or because of technical problems during data recording (psychophysiological measures).

The results of our statistical analysis agree with our expectations (see Table 1). First, we tested the contribution of aesthetic emotion components (autonomic, expressive, and subjective) on Aad formation. The level of arousal measured by both the self-reported measure (SAM; 1–5 scale, *M* = 1.8, SD = 0.856) and EDA (ISCR; M = 0.3831, Min = 0.28, Max = 1.41) was very low. As expected, the autonomic component (EDA) did not predict Aad (β = 0.047, *t* = 0.542, *p* = 0.589, ns). This finding aligns with the view that usual commercials elicit emotional reactions that are more aesthetic than utilitarian. Thereafter, only one of our two somatic measures of the expressive component (pleasure) showed that facial EMG influenced Aad. Z*ygomaticus major* activity had a significant positive effect on Aad (β = 0.279, *t* = 3.374; *p* = 0.00); however, *corrugator supercilii* activity did not appear to have a significant negative effect (β = -0.138, *t* = -1.667; *p* = 0.1). This finding is relevant considering the positive framing of commercials used in this study. Finally, the SAM indicated a positive effect of the subjective component on Aad (β = 0.534, *t* = 7.289; *p* = 0.00; Adj. *R*^2^ = 28%).

**TABLE 1:** Summary of the results.

	Direct effects of emotion components			
Independent variables	Dependent variable – Aad	Dependent variable – SAM Pleasure		Dependent variable – SAM Arousal
**Psychophysiological measures**				
EDA	β = 0.047, *t* = 0.542, *p* = 0.589	β = 0.045, *t* = 0.524, *p* = 0.601		β = 0.048, *t* = 0.562, *p* = 0.575
	*R*^2^_adjusted_ = 0.00	R^2^_adjusted_ = 0.00		R^2^_adjusted_ = 0.00
EMG_zygomatic	β = 0.279, *t* = 3.374; *p* = 0.00	β = 0.328, *t* = 4.41; *p* = 0.00		β = 0.179, *t* = 2.11; *p* = 0.04
EMG_corrugator	β = −0.138, *t* = −1.667; *p* = 0.1	β = −0.157, *t* = −1.964; *p* = 0.05		β = −0.056, *t* = −0.658; *p* = 0.512
	*R*^2^_adjusted_ = 0.09	*R*^2^_adjusted_ = 0.13		*R*^2^_adjusted_ = 0.02
**Self-report measures**				
Pleasure	β = 0.540, *t* = 7.375; *p* = 0.00			
Arousal	β = 0.095, *t* = 1.295; *p* = 0.198			
	*R*^2^_adjusted_ = 0.28			

	**Indirect effect between each measure of the expressive component on Aad**			

	EMG_zygomatic → Pleasure → Aad		Complementary mediation	
	EMG_corrugator → Pleasure → Aad		Indirect mediation	

Second, we tested the indirect effects of both the autonomic (EDA) and expressive (facial EMG) components of aesthetic emotion on Aad through the subjective component (SAM; Zhao et al., 2010). We did not observe any direct or indirect effects of the autonomic component (EDA) on either the subjective component (arousal measured through SAM) or on Aad. However, we observed that both measures of the expressive component (facial EMG) significantly predicted the pleasure dimension (SAM) of the subjective feelings component of aesthetic emotion (*zygomaticus major* activity: β = 0.328, *t* = 4.41; *p* = 0.00; c*orrugator supercilii* activity: β = -0.157, *t* = -1.964; *p* = 0.05; Adj. *R*^2^ = 13%). This result illustrates that the expressive component of aesthetic emotion (facial EMG) seems to be an antecedent of the pleasure dimension (SAM) of the subjective component. On the other hand, results indicated that facial EMG (only zygomaticus major activity) predict the arousal dimension (SAM) of the subjective feelings component as well (β = 0.179, *t* = 2.11; *p* = 0.04), which might be due to the intensity of the expressive component activation (Cacioppo et al., 1986). It offers psychophysiological validation and an objective foundation for this self-reported measure.

We then estimated the indirect effect between each measure of the expressive component (i.e., EMG activity of *zygomaticus major* and *corrugator supercilii*) and Aad (Preacher and Hayes, 2008; Zhao et al., 2010; Hayes, 2013). Based on a bootstrap analysis (95% confidence interval; CI), we first observed that the mean indirect effect between *corrugator supercilii* activity and Aad through the pleasure dimension (SAM) of the subjective feelings component was negative and significant (indirect effect = −3.4748) with a 95% CI that excluded 0 (−7.1628, −0.9245). In the indirect path, an increase of one unit in *corrugator supercilii* activity decreased self-reported pleasure by a = −1.406 (*p* = 0.04). An increase of one unit in self-reported pleasure also increased Aad by b = 2.47 (*p* = 0.00). However, the direct effect was just slightly above the usual and arbitrary threshold of significance (c = −6.19, *p* = 0.06). As a × b × c was positive and significant (*p* = 0.00), only an indirect mediation was at work. Second, we observed that the mean indirect effect between *zygomaticus major* activity and Aad through the pleasure dimension (SAM) of the subjective component was positive and significant (indirect effect = 3.8409) with a 95% CI that excluded 0 (2.1883, 6.1428). In the indirect path, an increase of one unit in *zygomaticus major* activity increased self-reported pleasure by a = 1.6472 (*p* = 0.00). An increase of one unit in self-reported pleasure increased Aad by b = 2.3318 (*p* = 0.00). The direct effect was significant (c = 6.61, *p* = 0.00). As a × b × c was positive and significant, there was complementary mediation at work.

These results reveal that the indirect effects of the expressive component of aesthetic emotion (facial EMG) on Aad are statistically significant. The pleasure dimension (SAM) of the subjective component of aesthetic emotion partially mediates the effect of facial EMG on Aad. Therefore, the self-reported and psychophysiological measures of aesthetic emotion are not interchangeable. Both are relevant to evaluate the impact of positive aesthetic emotions on Aad.

## What Does Aesthetic Emotion Mean for Investigating Consumer Emotions in Advertising Research?

In this perspective paper, we highlight the distinction between utilitarian and aesthetic emotions. To the extent that usual commercials do not seem to have obvious material effect on well-being and rarely lead to specific goal-oriented responses (e.g., fight or flight), the actual generated emotions—as shown by self-reported and psychophysiological measures—do not create important modifications in the autonomic nervous system that are largely devoted to behavioral readiness during utilitarian emotion. Therefore, our results encourage to focus on aesthetic emotions in advertising research.

Our results also shed new light on the respective contributions of the physiological, expressive, and subjective feelings components of aesthetic emotion elicited by usual commercials on Aad. Although researchers recognize the multi-component nature of emotions, most investigations have been restricted to the impact of the subjective component of emotion on Aad, with both elements being verbally measured. The current study departs from previous research by focusing on the respective contributions of autonomic, expressive, and subjective components of aesthetic emotions elicited by commercials in explaining Aad.

As expected, the autonomic component of emotion does not affect Aad, neither the physiological nor self-report measures. This might be due to the aesthetic nature of emotional episodes elicited by commercials. As stated by Mulligan and Scherer (2012, p. 353), the intensity of bodily reactions varies “and some emotions, such as […] aesthetic emotions, may have much subtler bodily manifestations than the utilitarian, survival emotions such as fear, anger, or disgust.”

Contrary to the arousal level of aesthetic emotion, the somatic (expressive component) as well as the self-reported (subjective component) levels of pleasure positively influence Aad. This result aligns with our assumption that aesthetic emotions are primarily derived from the appreciation of “the intrinsic qualities” of the stimulus (Scherer, 2004, p. 244) that contribute to the formation of consumer attitudes. The subjective feelings of pleasure partially mediate the effect of facial EMG on Aad (i.e., the subjective component of pleasure partially rests on expressive motor reactions). Therefore, self-reported emotions may reflect actual emotional episodes better than a simple manifestation of social desirability or the use of display rules that voluntarily alter affective reactions. By dealing with aesthetic emotions in the case of commercials (Scherer, 2004), we determined that subjective feelings of pleasure/displeasure represent genuine perceptions according to their facial EMG measurement and partially mediate the effects of facial EMG on Aad. In this exploratory research we confirmed the sequential perspective proposed by Scherer (1984) by showing that the expressive (motor) component precedes and explains the subjective feelings component of emotion.

On a methodological note, our results highlight the automatic and corresponding background of pleasure assessed iconically (i.e., SAM scale). The consideration of both sides of the same coin led us to validate an iconic measure of pleasure. However, we did not find similar correspondence or consistency in the case of arousal, presumably because we registered particularly low levels of autonomic arousal (through EDA) for aesthetic emotions. Therefore, facial EMG might be more informative than EDA for measuring aesthetic emotions in advertising research. In addition, this research confirms that self-reported and psychophysiological measures are more complementary than mutually exclusive.

Finally, an important implication that emerges from the results of this research is that special attention must be paid to the merits of each method. For example, to estimate ad effectiveness, communication agencies and neuromarketing companies often use devices like a wristwatch that measures arousal given this device’s ease of use. However, the current research shows that the evaluation of usual ads mainly relies on pleasure, not on arousal. So, special attention must be paid to their ability to induce pleasure when pretesting usual ads.

## Limitations and Future Directions

This perspective paper is exploratory, and its results must be tempered to some extent by limitations that deserve to be addressed in further research.

First, we use a limited set of only three commercials. Replications with less sales-oriented commercials (e.g., cause-related) could reveal how generalizable our results may be. Replications with more engaging products (e.g., cars, luxury goods) would also be useful for testing the robustness of our findings. Moreover, it is possible to elaborate a strategy aiming to elicit instrumental behavior through emotional reactions using “endorsers” describing how good they feel after quitting their “smoking addiction”, or how irrational they were to keep on smoking too many years with severe health problems as a consequence. Nowadays there are campaigns to adopt behavioral measures (e.g., social distancing and/or wearing a mask) to protect people against COVID-19. However, this type of advertising campaigns is more the exception than the rule.

Second, it is widely accepted that the face is central to a system of rapid, emotion-revealing signals, and arousal is often described as a key dimension of an emotion. Using physiological tools (facial EMG and EDA) requires neither retrospection nor introspection. However, the tools used in this study are extremely difficult to implement in a naturalistic environment. To advance toward ecological validity, forced exposure must be reduced by using a real program in which commercials are embedded.

Third and finally, this research focuses on affective independent variables. In the future, it may be important to integrate variables of a more cognitive or evaluative type—including recall, recognition, or evaluation of the arguments and elements of ad execution to attain a more comprehensive view of the topic. Focusing on the elements of execution of the commercial seems particularly essential to the extent that, as stressed by Scherer and Zentner (2008, p. 596) “in the case of aesthetic emotions appraisal tends to be more intrinsic to the visual or auditory stimulus, based on forms and relationships.”

## Author’s Note

This research relies on ML’s Ph.D. dissertation.

## Data Availability Statement

The raw data supporting the conclusions of this article will be made available by the authors, without undue reservation.

## Ethics Statement

The studies involving human participants were reviewed and approved by École Universitaire de Management de Rennes. The participants provided their written informed consent to participate in this study.

## Author Contributions

ML conceptualized the study, set up the psychophysiological study, processed the physiological signal, performed descriptive statistics, wrote the draft, and revised the final draft. OD was the supervisor of the Ph.D. thesis on which this research relies and contributed to all the stage of the development of the present research. CD and IP contributed to the conceptualization of the study, performed the statistical data analysis, and participated to improve the manuscript. All authors contributed to the article and approved the submitted version.

## Conflict of Interest

The authors declare that the research was conducted in the absence of any commercial or financial relationships that could be construed as a potential conflict of interest.

## Footnotes

1 Considering the technical constraints related to setting up an electrophysiological recording device (e.g., time spent to equip participants, cost of consumables), the size of our sample is not directly comparable to the size of samples of more common quantitative methods, such as those using questionnaires. A review of previous psychophysiological marketing research shows that the average sample size of studies similar to ours is 45 participants (Droulers and Lajante, 2015).
